# Attitudes and Practices of Australian Veterinary Professionals and Students towards Early Age Desexing of Cats

**DOI:** 10.3390/ani9010002

**Published:** 2018-12-20

**Authors:** Heather M. Crawford, Michael C. Calver

**Affiliations:** Environment and Conservation Cluster, School of Veterinary and Life Sciences, Murdoch University, Perth 6150, Australia; crawfh01@gmail.com

**Keywords:** cat, early age desexing, education, legislation, nurse, student, survey, veterinarian, website

## Abstract

**Simple Summary:**

Globally, desexing is used to reduce/prevent overpopulation of cats. However, its effective use is hampered by widely held views that it should occur at around six months of age, whereas female cats can breed from four months of age. As a result, many cats may have an unplanned litter before they are desexed. In Australia, increasing numbers of municipalities are mandating desexing of cats by three months of age, before the traditional age of 4–6 months. Achieving this goal requires support from veterinary professionals, so we used online and face-to-face surveys to determine the preferred desexing ages for cats and rationale of 957 Australian veterinarians, veterinary nurses, veterinary science students, and veterinary nursing students. A complementary survey of 299 veterinary practice websites across Australia documented information provided about desexing cats. Vet nurses and nursing students were more conservative than vets or vet students, preferring to desex cats (especially females) after four months because of concerns about anaesthetic risk. Over half of surveyed websites provided no information about desexing cats or offered desexing without explaining why it was necessary or when to perform it. In Australia, the preferences and practices of some current/future veterinary professionals do not match changing cat legislation.

**Abstract:**

Surgical desexing of cats is typically carried out after six months of age (Mature Age Desexing, MAD); between 4–6 months (Traditional Age Desexing, TAD); or before four months (Early Age Desexing, EAD). We complemented existing surveys of veterinarians’ acceptance of EAD with online and face-to-face surveys, to ascertain the preferred desexing ages for cats and rationale of 957 Australian veterinarians, veterinary nurses, veterinary science students, and veterinary nursing students. A complementary survey of 299 veterinary practice websites across Australia documented any information provided about desexing cats. The most common reason for preferred desexing ages was reducing stray cat populations (30%); 78% of these respondents chose ages aligning with EAD. Vet nurses and nursing students were more conservative than vets or vet students, preferring to desex cats >4 months. Perceived anaesthetic risk was a major motivation, especially for nurses ≤5 years’ experience. Across 299 urban practices in Australian capital cities, 55% of surveyed websites provided no information about desexing cats or listed desexing without explaining why it was necessary, or when to perform it. Increasingly, Australian legislatures mandate desexing of cats by three months of age, so the practices of some current/future veterinary professionals do not match changing legislation.

## 1. Introduction

Globally, domestic cats (*Felis catus*) entering animal shelters are more likely to be euthanized than dogs (*Canis familiaris*) [1,2,3,4]). Over the past decade, approximately 57% of cats entering Royal Society for the Prevention of Cruelty to Animals centres across Australia have been euthanized (RSPCA [5]), while estimates from the United States of America (69% in Ohio, USA [6], and up to 71% in West Coast States ([7]), Canada (53% in Ontario [8]), and the United Kingdom (13% [3]) indicate that high euthanasia rates are of international concern. In Australia, most cat admissions to shelters are unwanted litters and strays, 87–90% of which are sexually intact [9,10,11,12]. Constant, high euthanasia rates present an ethical conundrum for animal welfare and the mental health of shelter employees/volunteers [13,14,15,16,17]. Cats are increasingly popular pets [18,19,20], comprising 30% of all pets visiting private veterinary practices in the UK [21]. In Australia, 29% of households own at least one cat [22], and cats and dogs make up 88% of the total workload in private practices [23]. As providers of medical treatment, surgical procedures and chemical euthanasia, professionals in the veterinary industry are key stakeholders in facilitating population control and reducing euthanasia rates of cats [24,25,26].

One of the principal tools used to reduce cat populations is surgical desexing (ovariohysterectomy and gonadectomy). Performed by registered veterinarians (vets) and aided by veterinary nurses, desexing is typically performed when animals are older than six months of age: (Mature Age Desexing, MAD); when animals are 4–6 months of age (Traditional Age Desexing, TAD); or when animals are younger than four months of age (Early Age Desexing, EAD) [27,28]. TAD and MAD are routine in private veterinary practices EAD is typically performed in shelters to prevent future breeding and to expedite adoption [27,28]. Professionals in private practices may practice EAD less frequently depending on their personal experience and owner preferences [29,30].

The disparity in desexing age between shelters and private practices may exacerbate unwanted cat populations. Even a few sexually intact animals can destabilise efforts to control populations (cats produce average litters of 3–4 kittens, 2–3 times per year depending on breed, physical condition, presence of sexually intact male cats, number of daylight hours, and hemisphere [31]). While some cat owners never desex their cats, others allow cats one litter because they believe this is healthy [32,33,34,35], although this is not supported in the literature. Additionally, approximately 70.5% of litters are unplanned because owners are unaware that their cat is reproductive [32,33,34,35,36]. Cat owners may be unaware that females can reproduce as young as four months and may not recognise behaviours associated with puberty and receptiveness in females (continuous reproductive cycling with induced ovulation and no vaginal discharge, increased affection, and express lordosis; [37]) compared with female dogs (single season with spontaneous ovulation, swollen vulva and vaginal discharge [37]). Male cats mature sexually at 8–12 months of age and can mate throughout the year [37]. Consequently, owners may only seek professional advice about desexing when cats are already pregnant [38]. Owners who are unwilling, or unable, to care for and rehome the litter may abandon or surrender the cats [34,39].

Despite the oversupply of unwanted litters and stray cats, many Australian veterinarians favour either TAD or MAD (32% and 49% respectively) as they personally recognise little need to reduce the desexing age in either cats or dogs [40,41]. Leung et al. [41] found that although 99% of surveyed veterinarians (*n* = 774) believed desexing should be routine for non-breeding cats (e.g., pedigree cats), younger veterinarians were more likely to recommend ≥6 months of age, and older vets more willing to desex younger cats. An earlier survey of opinions toward EAD in cats found that 63% of Australian vets thought desexing before puberty was desirable (*n* = 174), yet the mean age of puberty across both sexes was estimated to be 5.8 months [40]. It therefore, appears that accidental litters amongst pet cats are high because of naivety in owners [33,42], and possibly because veterinarians provide conservative advice about when puberty occurs in cats [43]. Promoting and increasing uptake of EAD in private practices will remove the onus on cat owners to identify puberty and facilitate high desexing rates that are stable over time and across pet cat populations.

While the attitudes of veterinarians have been studied extensively [30,40,43] and some data are now available on the attitudes of veterinary teaching staff [44], private practices and shelters also employ veterinary nurses and offer work placement to students of veterinary science and veterinary nursing. The attitudes of these latter groups are unknown, yet they assist in surgery, perform clinical tasks, and interact with the owners enquiring about desexing of various species and breeds [24,26,45,46]. Student attitudes are shaped by their education and experiences, so their opinions on minimum desexing ages may identify areas for reform within curricula. Jupe et al. [44] found that only nine of 40 staff (22.5%) at eight universities in Australia and New Zealand personally advocated the use of EAD for male or female cats, and that very few students witnessed or practiced EAD before graduating. With limited exposure to EAD it is unlikely that students will recommend it to private clients.

This two-part study extends the previous studies of veterinarian attitudes towards desexing in Australia [40,41,44], by assessing the opinions of veterinary nurses, veterinary science students, nursing students and a small group of veterinarians about minimum desexing ages for male and female cats.

Part 1 used online surveys and face-to-face interviews to determine whether:

1. There is a preferred minimum desexing age for male and female cats;
2. Preferred minimum desexing ages are chosen for particular reasons; and
3. Desexing ages and reasons are influenced by professional role or career length within the veterinary industry; and
4. Respondents concerned about overpopulation favoured EAD.

Part 2 used a desktop survey of 299 private veterinary practice websites in capital cities of Australia’s six states and two territories to document what information is provided to the public about desexing cats. The aims of Part 2 were:

1. To determine and compare the desexing ages recommended for cats by urban veterinary practices in different Australian states/territories;
2. To compare results for cats with desexing information presented for dogs; and
3. Identify whether the desexing ages recommended by practices comply with any state/territory mandatory desexing legislation.

## 2. Materials and Methods 

### 2.1. Part 1—Design and Administration of Surveys Assessing Preferred Ages for Desexing Cats and Reasons for These Preferences

Surveys were authorised by the Murdoch University Human Ethics Committee (permit number: 2014/099) and conformed to the National Statement on Ethical Conduct in Human Research [47]. Surveys were anonymous and designed to take no longer than 10 minutes. Respondents were not directly surveyed about EAD; instead, neutral language was used to elicit preferred minimum desexing ages. Surveys comprised three sections: demographic information of respondents, male cat desexing age with rationale for preference, and female cat desexing age with rationale for preference (Appendix A, Table A1). Demographic information consisted of gender, profession (veterinarian, veterinary nurse, veterinary student, veterinary nurse student or other), length of career in years (subsequently grouped into the categories of <1 year, 1–5 years, 6–10 years, 11–15 years, 16–20 years, and >21 years), postcode of current or last placement at a veterinary practice, and affiliation with professional organisation/s (Australian Veterinary Association, AVA [48]; and Veterinary Nurses Council of Australia, VNCA [49]). Respondents could choose from five pre-determined preferred minimum desexing age ranges or could specify a different age for either cat sex. Respondents were then asked to justify their minimum age by selecting ≥1 of five pre-determined reasons, or they could specify their own reasons. Extra information volunteered by respondents was further categorised (e.g., desex cats for a shelter, practice offering kittens for adoption).

Initially, face-to-face surveys were carried out at the conference of the Western Australian chapter of the AVA in May 2014; and at the VNCA’s National Conference in April 2015. These confirmed that the survey design was clear to a wide range of potential respondents and that no modifications were needed before offering the survey online. The seven Australian universities offering a degree in veterinary science were then contacted to invite students in their final two years of study to complete an online version of the EAD survey (hosted by SurveyMonkey Inc. [50]). Four universities agreed to distribute the survey link in October 2015 and 2016 (Murdoch University, Western Australia (WA); The University of Sydney and Charles Sturt University, New South Wales (NSW); and The University of Melbourne, Victoria (VIC)). The same invitation was extended to 14 private or State institutes offering certificates in Veterinary Nursing. Nine institutes distributed the survey link in October 2014 and 2015 (WA: Applied Vocational Training and Polytechnic West. NSW: New England, North Coast, Sydney and Western Sydney Institutes of Technical and Further Education. VIC: Box Hill Institute and Melbourne Polytechnic. Queensland (QLD): Technical and Further Education Queensland). Students were emailed the survey link by staff at participating institutions. The total number of students who received email surveys was unknown to researchers, therefore, no response rate could be calculated.

### 2.2. Part 2—Desktop Survey of the Desexing Ages Recommended by Veterinary Practices

In 2016, a desktop survey was carried out by searching for cat desexing information on the websites of 50 urban veterinary practices in the capital cities of four Australian states—Sydney (NSW), Melbourne (VIC), Brisbane (QLD), and Perth (WA). At the time of survey, only one of the surveyed States—WA—had cat-specific legislation mandating desexing of cats by six months [51]. 

In 2018, the survey was extended to include Australia’s two remaining states and two territories: Adelaide (South Australia, SA), Hobart (Tasmania, TAS), Canberra (Australian Capital Territory, ACT), and Darwin (Northern Territory, NT). In 2000, the ACT had mandated cat desexing by six months of age and this was lowered to three months in 2014 [52]. TAS introduced mandatory desexing for cats by six months in 2009 [53] and desexing by six months applied in SA from 2018 [54]. As yet, NSW, VIC, QLD, and the NT do not have state-wide legislation that mandates desexing of cats. Therefore, when the desktop survey was extended in 2018, the ACT and TAS had had desexing legislation in place for several years, and SA was due to implement legislation introduced in 2017. 

The name of one capital city at a time and *“veterinary”* were typed into Google Maps [55]. Moving concentrically out from the city Central Business District (CBD), the first 50 veterinary clinics/hospitals/centers with operational websites were surveyed. The small populations of Hobart, Canberra and Darwin (<250,000, <400,000, <107,000 people, respectively [56]), meant that <50 websites existed, so fewer were surveyed. Websites were searched for any information on desexing (search terms included: *“desex(ing); breeding; sterilis(z)ation; surgery; puberty; reproduce(ing/tion)”*). Some websites had a section outlining desexing surgery, while other sites provided a link to the same generic template that provided more detailed information about the advantages and disadvantages of desexing generally (based on information from the USA). Where websites and/or linked templates mentioned ages/age ranges for desexing either male or female cats, these details were recorded. For comparative purposes the same information was collected for pet dogs.

NSW was the first State searched and its results were used to fine-tune the searches of other States. Veterinary practices belonging to chains were initially surveyed but then excluded to prevent over-representing desexing recommendations because all practices recommended the same/or no desexing ages, often using the same generic template. Practice chains are increasingly popular in Australia, so their recommendations for desexing ages are relevant to this study and although not analysed, practice chain data are included in Appendix B (Table A2). Shelter groups and their associated veterinary practices were also initially surveyed but then excluded as TAD and EAD were standard and would also overweight responses (Appendix B, Table A2). Specialist centres (e.g., emergency, orthopaedics) did not appear to offer the public standard veterinary services and were excluded. Practices included in the desktop survey were, therefore, privately owned, small or mixed-animal practices offering standard and some specialist services to the public.

### 2.3. Part 2—Data Analysis

In all χ^2^ analyses described below, we followed the recommendation of Zar [57] that the minimum mean expected value in contingency tables be ≥6 for testing at a significance level of 0.05. χ^2^ analyses were carried out using the online resource VassarStats [58].

For vets, preliminary χ^2^ contingency table analysis showed no association between mode of data collection (face-to-face vs. online surveys) and response choices for preferred desexing ages for male (χ^2^ = 6.7, d.f. = 3, *p* = 0.08) or female cats (χ^2^ = 4.6, d.f. = 3, *p* = 0.21) if the category ‘other’ desex age was excluded. For vet nurses, there was no association between mode of data collection and preferred desexing ages for male cats (χ^2^ = 7.6, d.f. = 3, *p* = 0.06) with the ‘other’ category excluded, but for female cats they were significantly more conservative in their age choices when surveyed face-to-face (χ^2^ = 15.4, d.f. = 3, *p* < 0.01). Face-to-face numbers for vet students, nurse students, and ‘other’ professionals were too small for valid comparisons with online numbers. Given that the category ‘other’ is described independently in all subsequent analyses to allow for the diversity of responses included in this category and that only the responses from nurses for female cats differed between survey method, data from the online surveys and the face-to-face interviews for the different professional roles were combined to increase sample sizes for all further analysis (see Appendix C for data).

With regard to survey demographics, if survey respondents selected >1 option for profession (e.g., registered veterinary nurse currently enrolled in veterinary science), their choices of preferred desexing age and reason for male and female cats were counted twice, once per professional category.

With regard to rationale for preferred minimum desexing ages, where respondents selected >1 reason then answers were weighted by dividing by the number of categories chosen. For example, if a respondent chose two reasons, the frequency would be scored as 0.5 in each of the two categories. Key comments made by respondents that were strong statements of attitudes, practices or misconceptions, were listed.

Associations between profession, career length and the preferred age of desexing for male and female cats; or the reasons for selecting specific ages, were assessed with three-way log-linear contingency tables using VassarStats [58], incorporating a sequential Bonferroni correction for the multiple hypotheses. Where respondents chose the ‘other’ option rather than a specified category for desexing age or reason, these responses were summarised with descriptive tables and graphs. χ^2^ contingency tables were used to test whether or not the choice of multiple reasons for choosing a desexing age varied between different professions.

Data from the desktop survey (Part 2) were described and presented without statistical analysis.

## 3. Results

### 3.1. Part 1: Preferred Ages for Desexing Cats and Reasons for These Preferences

#### 3.1.1. Characteristics of Survey Respondents

Between 2014 and 2016, 209 face-to-face surveys were carried out and 777 online surveys were received (*n* = 986; Table 1). Respondents were mostly veterinary nurses, veterinary students, and veterinary nurse students, with smaller cohorts of veterinarians and respondents with ‘other’ roles in the industry (mostly educators of veterinary science and nursing subjects; Table 1 and Table 2). Forty-four respondents (5%) selected >1 category for profession (e.g., veterinary nurse was also a student enrolled in veterinary science).

While it was not possible to calculate response rates for online surveys because we do not know how many people were invited to participate, only 47 online respondents logged-out of the survey mid-way and did not return (6%, *n* = 777 online respondents). These surveys and those of 16 non-professionals (e.g., clerical staff at educational institutions) and 10 respondents who did not specify a profession, were excluded from all subsequent analyses (7%, *n* = 73/986, new total *n* = 957).

The three survey sections were answered in full by 587 respondents (61%, *n* = 957), leaving 370 partially completed surveys. Section 1 questions (respondent demographic information) were answered in full by 66.5% of respondents. Section 2 questions (preferred minimum desexing age for male cats with reasons) were answered by 87% of respondents, and questions in Section 3 (preferred minimum desexing age for female cats with reasons) were answered in full by 85%. Of all survey questions, respondents were least likely to provide a postcode for their place of work (67%, *n* = 957).

Forty-two percent of respondents were yet to practice in the industry, and nearly 30% had been practicing for 1–5 years (Table 2). Forty-eight vet students and 114 nurse students had acquired unqualified experience in the veterinary industry (>0 years). Respondents were mainly women (99.8% vs. 0.2% men), with 55% belonging to one, or both, official industry organisations (AVA or VNCA). Postcode data indicated that most respondents currently worked/previously worked in either WA (36%, *n* = 600), or NSW (23%), and probably reflected the state of residence of respondents. Twenty respondents worked/had worked overseas (3%, *n* = 600; Hong Kong, Ireland, Kuwait, New Zealand, Singapore, South Africa, Timor Leste, UK, and USA).

#### 3.1.2. Minimum Desexing Age for Male Cats

Pre-determined desexing ages were selected by 761 survey respondents (79.5%, total *n* = 957; Table 3), with the most popular choice for veterinary nurses and nurse students being >4 months, which was older than the ages chosen by the other professional cohorts. A further 69 respondents selected ‘other’ ages (7%; Table 4). These respondents were mostly vet nurses (74%, *n* = 51) and tended to provide *“if/or”* answers (e.g., *“desex male IF presented to vet at 12 weeks of age OR from 1.0 kg—whichever comes first”*). Most ‘other’ respondents were comfortable using body weight of male cats to guide timing of desexing (30%, *n* = 21). Another small group thought that minimum desexing age should vary depending on whether cats were clients at private veterinary practices or were entering/adopted from animal shelters (27.5%, *n* = 19—typically favouring >4 months for private practice cats *OR* ≥1.0 kg for cats in shelters). No minimum desexing age was specified by 127 respondents (13%), most of whom were veterinary students and probably reflects some uncertainty on the part of students.

#### 3.1.3. Minimum Desexing Age for Female Cats

Pre-determined desexing ages were selected by 751 survey respondents (78.5%, total *n* = 957; Table 3), a slightly lower frequency than observed for male cats. The most commonly selected category for veterinarians was 3–4 months, while for all other professional roles chose >4 months. A further 65 respondents selected ‘other’ ages (7%; Table 4). These respondents were mostly vet nurses (71%, *n* = 46) and tended to provide *“if/or”* answers (e.g., *“desex female IF presented to vet before first season OR from 2.0 kg”*). Most ‘other’ respondents thought that minimum cat desexing should vary depending on whether cats were clients at private veterinary practices or were entering/adopted from animal shelters (34%, *n* = 22—typically favouring >4 months for private practice cats *OR* ≥1.0 kg for cats in shelters). More respondents specified no minimum desexing age for female cats than males (15%, *n* = 141 vs. 13%, *n* = 127). Most of these respondents were veterinary students, and again probably reflects some uncertainty on the part of students.

#### 3.1.4. Rationale for Minimum Desexing Ages for Male Cats

To justify choice of minimum desexing age for male cats, pre-determined reasons were selected by 831 survey respondents (87%, *n* = 957; Table 5). No reasons were provided by 126 respondents (13%). Weighted responses showed that reducing the number of stray cats in the community motivated 30.5% of respondents’ choice of preferred minimum desexing age for male cats. ‘Other’ reasons for choice of preferred minimum desex age were provided by 140 respondents (14.5%; Table 6), most of whom were vet nurses (65.5%). The most popular ‘other’ reason for choice of minimum age was pre-empting general problem behaviours/sexual maturity (25%, e.g., fighting, roaming). Thirteen percent of respondents did not provide reasons for their choice of desex age (*n* = 126), 64% of whom were vet students—possibly indicating some uncertainty in students. Approximately equal proportions of vets, vet nurses, and nursing students selected single or multiple reasons for their choice of minimum desexing age for male cats (Table 7). More ‘other’ professionals selected a single reason for their choice of desexing ages, whereas more vet students selected >1 reason for their choice of ages, which again may signify some uncertainty. Overall, this led to a significant association between the number of reasons chosen to justify minimum desexing age for male cats and the different professional roles of respondents (χ^2^ = 92.8, d.f. = 8, *p* < 0.0001).

#### 3.1.5. Rationale for Minimum Desexing Ages for Female Cats

For female cats, 814 survey respondents selected >1 pre-determined reasons to justify their choice of minimum desexing age (85%, *n* = 957; Table 5). No reasons were provided by 143 respondents (15%). Weighted responses showed that reducing the number of stray cats in the community motivated 30% of respondents’ choice of preferred minimum desexing age for female cats. ‘Other’ reasons for choice of minimum desex age were selected by 130 respondents (13.5%; Table 6), who were mainly vet nurses (69%). The most popular ‘other’ reason for choice of minimum age was following practice protocol/being guided by veterinarians’ surgical skills and wishes (21%). A slightly higher percentage of respondents provided no reasons for their choice of female desex age compared with male desex age (15%, *n* = 143 vs. 13%, *n* = 126). Most respondents providing no rationale were vet students (63%)—possibly indicating some uncertainty on the part of students. Single reasons to justify choice of preferred minimum desex age were selected by more vets, vet nurses, and ‘other’ professionals—compared with both student cohorts who selected >1 reason (Table 7), again possibly indicating some uncertainty about the appropriate desexing age for female cats. Overall, this led to a significant association between profession and the number of reasons chosen to justify minimum desexing age for female cats (χ^2^ = 106.3, d.f. = 8, *p* < 0.0001).

#### 3.1.6. Support for EAD by Respondents Concerned about Overpopulation

Approximately 78% of survey respondents who were concerned about the number of stray cats in the community chose desex ages for male cats that align with EAD (*n* = 516; Table 8). Fewer respondents selected EAD for female cats despite their concerns about overpopulation (61%, *n* = 406; Table 8). Fifty-percent or more of respondents who were concerned about anaesthetic risk, their personal lack of experience desexing younger animals, and abnormal development following desexing, none-the-less selected EAD for male cats. More respondents with the same concerns chose TAD/MAD for female cats (44.5% to 55%).

#### 3.1.7. Key Comments from Survey Respondents

Many survey respondents provided additional comments to explain their choice of minimum desexing ages. There was great variability in respondent attitudes about if, when, and why cats should be desexed. Key comments listed below illustrate the importance of ensuring that professionals and students have accurate knowledge of cat biology, increased experience with EAD and that practice ‘norms’/protocol are updated. Inclusion of a comment is to illustrate the range of opinions and implies neither factual accuracy nor our endorsement of any position.

Veterinarians:

*1.*  I use body weight of 1.0–1.2 kg as there is no medical/surgical evidence for a ‘correct’ desexing age. Entirely dependent on surgeon’s abilities but heat loss can be an issue depending on body surface area.
*2.*  It is easier to rehome kittens at 2–3 months which then makes it cheaper for vet/shelter as otherwise they must house and feed cats until they’re older.
*3.*  Desex at 3–4 months as kitten should now be fully vaccinated and well settled in his/her new home.
*4.*  Desex at last kitten vaccination as many cats are not seen after this or before they are pregnant.
*5.*  See no need to do earlier than 3–4 months, and it’s easier to give I/V drugs and intubate after this age.
*6.*  Testicles are very small before 3–4 months and the surgery is therefore, more prone to error.
*7.*  At 9–12 weeks recovery from surgery is fastest, less weight is gained, and it is now law.
*8.*  Older than 4 months as younger cats’ anatomy is difficult to work with.
*9.*  Easier surgery from 2–3 months. I don’t understand the resistance to EAD. Chose minimum desex age based on data not tradition.
*10.*  Lots of breeding cats is good as it means more money for vets.

Veterinary Nurses:

*1.*  Desex at 2–3 months as I have worked in a shelter and we need to control numbers and dispel the misconception that cats should have one litter.
*2.*  Recommend desexing from 1.0 kg—there are urinary tract issues but I don’t care, it’s more important to stop breeding.
*3.*  Recommend desexing from 800 g as saw mortality rates were low when working for charity.
*4.*  Surgery is less complicated in males and recovery is quick, so I would do younger than 6 months if asked by owner of practice.
*5.*  Desex at 3–4 months as their recovery from surgery seems much better than older cats.
*6.*  There are kittens brought in by registered breeders that choose to desex (at 3–4 months) before they go to new homes, otherwise we recommend 5–6 months of age.
*7.*  Desex from 7 months as bodies are mature but bad behaviours do not yet exist.
*8.*  Would prefer to desex after 9–12 weeks as female cats put on lots of weight.
*9.*  Desex after 4 months as I have seen poor joint development in kittens desexed at younger ages.
*10.*  Desex after 6 months when two testes are descended.
*11.*  Desex from 2–3 months as male cats revert back to a wild state when hormones take over. I’ve only met a few nice entire males. They become aggressive, they fight, smell, spread FIV, and get hit by cars.
*12.*  I was taught desexing at 3–4 months of age and comfortable with it, but practice protocol where I work is 5 months.
*13.*  The vet I work with is very experienced with young surgeries, so I am comfortable following his wishes.
*14.*  We desex from 8 weeks as it is the vet’s preferred age because surgery is more fiddley when older.
*15.*  Desex from 1.0 kg as I work in a low-income area so need to desex as soon as possible as people don’t return after vaccinations are finished.
*16.*  Our clinic packages a cat’s last vaccination with desexing at 4 months otherwise clients don’t come back.
*17.*  Don’t like to desex before 3 months as you can’t use appropriate pain relief for them.
*18.*  Owners get upset if you suggest desexing their animals when they are still small.
*19.*  Desex after 6 months as I hate neonatal surgery and I want organs to be more developed.
*20.*  The practice where I work is owned by a vet who refuses to desex under 6 months. (He/she) will write a medical certificate for pet owners to give to the council so they won’t get fined for not desexing earlier.
*21.*  Desex after 4 months as I believe that the ‘new’ thinking in favour of EAD is wrong. Those practicing EAD should be charged with cruelty and struck off the registry.

Veterinary Students:

*1.*  Males should be desexed at an older age than females.
*2.*  I have not come across desexing patients younger than 4 months.
*3.*  I prefer 5–6 months but it also depends on the size of the cat and if owners will keep them inside or outside.
*4.*  Desexing after 4 months allows full development of the urinary tract.
*5.*  In an ideal world we would desex at 2–3 months. Desex younger if owner is unlikely to bring them back later.
*6.*  It’s not an age thing for me, weight is more important.
*7.*  Weight should be taken into consideration—if a kitten reaches 5 months but is not a healthy weight the surgery should not be carried out.

Nurse Students:

*1.*  Desex after 6 months as testicles will have dropped.
*2.*  Desex after 5 months as we like to check for deciduous teeth.
*3.*  We have only been advised that the optimal age is 6 months, but I know that charities spay and castrate from 800 g.
*4.*  I feel it the owner’s call as to what they feel comfortable with.

#### 3.1.8. Influences of Professional Role and Career Length on Preferred Age of Desexing and the Reasons Given

The profession of survey respondents significantly affected their preferred minimum desex ages for both male and female cats (G ^2^ = 203.60, d.f. = 40, *p* < 0.0001; Table 9, Appendix D). Nurses and nurse students were more conservative than vets or vet students, preferring to desex both male and female cats >4 months. Career length did not affect the preferred desex ages chosen by professionals or students. Two-way interactions revealed that vet students and vet nurses preferred to desex female cats at older ages than male cats.

The profession of survey respondents also significantly affected the reasons chosen to justify their choice of preferred minimum desex ages for male and female cats (G^2^ = 201.64, d.f. = 40, *p* < 0.0001; Table 10, Appendix E). Veterinarians and students of both disciplines mostly chose desexing ages they believed would lead to reduced stray cat populations for both cat sexes; vet nurses were more motivated in choice of desexing age for female cats by anaesthetic risk. Career length did not affect the reasons chosen by veterinarians or student cohorts. However, two-way interactions revealed that nurses with ≤5 years of experience were most motivated by anaesthetic risk whereas nurses with >5 years of experience were motivated by desire to see reduction in stray populations.

### 3.2. Part 2—Desktop Survey of the Desexing Ages Recommended by Veterinary Practices

Desktop surveys of websites belonging to 299 urban veterinary practices in the capital cities of Australia’s six states and two territories, found 133 websites made no mention of desexing cats whatsoever (including two holistic veterinary practices offering no surgical services; Table 11). Thirty-one practices listed cat desexing as a service but recommended no specific or minimum age/age range (total with no desexing information *n* = 164). Similarly, 130 practice websites made no mention of desexing dogs, while 28 listed desexing dogs as a service, but with no recommended age/age range (total *n* = 158). Two websites specifically mentioned desexing of cats but not dogs (0.7%), and eight websites mentioned desexing of dogs but not cats (3%). A single practice offered specialist fertility services for breeders of cats and dogs, and another practice promoted contraceptive drugs and implants because: *“There is an increasing amount of information in mainstream, peer reviewed journals that highlights the problems with desexing and the undesirable outcomes dating back to the 1970s”.*

Fewer than half of surveyed veterinary practice websites specified a desexing age for cats (45%, *n* = 135/299; Table 11). Of these, 93% recommended the same age/age range for desexing regardless of cat sex (*n* = 125; Table 12). When recommended ages were grouped into desexing categories using the lowest listed ages (i.e., EAD, TAD, MAD, plus ‘general statements’), it was clear that practices most frequently recommended TAD for both cat sexes (91%, *n* = 125/135; Table 12 ). The remaining websites that provided information about when to desex cats (7.4%, *n* = 10/135, three websites specified ages for female cats only and made no mention of males), also recommended TAD more frequently than other desex age categories (six of seven websites with male ages, five of 10 websites with female ages).

Similarly, for dogs, only 47% of practice websites specified a desexing age (47%, *n* = 141/299; Table 11). The same age/ranges were recommended for desexing both male and female dogs by 94% of websites (*n* = 132/141; Table 12). A total of nine websites differentiated between when to desex male and female dogs (five websites made no mention of male dogs, providing ages for females only). Ages categorised as TAD were most frequently recommended for dogs of both sex (95%, *n* = 125/132), and even for individual sexes (three of four websites with male ages, four of nine websites with female ages).

Nearly 45% of practice websites surveyed specified a desexing age/age range for both cats and dogs (*n* = 133/299; Table 11). Most of these websites recommended desexing both species at the same age/age range (87%, *n* = 116; Table 13). Ages classified as TAD were most frequently recommended for both species.

## 4. Discussion

Veterinary professionals in many, but not all, countries favour surgical desexing to control cat populations because it is permanent, confers behavioural and health benefits [59,60], and reduces the social costs of unowned animals [3,17,61]. Desexed cats suffer fewer fighting injuries, display less aggression, have reduced incidences of tumours of the reproductive tract, seldom exhibit sexual behaviours such as spraying, and have longer life expectancy [62,63]. Contraceptives and drugs to delay puberty are being trialled where there are ethical or welfare objections to surgery [64,65], but they can be costly are impermanent, and provide few, if any, behavioural or health benefits. However, our study shows that there is no consensus amongst Australian veterinary professionals or students on the ideal age to desex pet cats, with limited, conservative advice available online to clients via practice websites. Similar situations occur elsewhere (e.g., the UK [61]). The reasons are complex, with changing social expectations and slow uptake of research evidence probably important. Nevertheless, changing legislative contexts in Australia reflect a public wish for mandatory desexing of cats, with implications for practicing veterinary professionals and veterinary education.

### 4.1. History of Practice and Evidence Regarding EAD

The preferred age to desex pet cats has varied with time. In the early 20th century, female cats were desexed between three and six months of age, while males were castrated as young as four weeks [66]. Now, TAD and MAD are routinely carried out in private practices, and EAD is common in shelters (e.g., [30]). The shift to later ages for desexing in private practices may reflect a concern to minimise perceived risk to emotionally and financially valued pets [66].

Since the early 21st century, several studies have addressed objections to EAD for cats [27]. They indicated that anaesthetic risks and surgical complications were less, or no greater, for EAD cats than for older cats [67,68], and that post-operative complications are low (e.g., incision-site infections [69,70]). Desexing at, or near, the time of vaccinations did not inhibit antibody response [71,72]. Abscesses, aggression, sexual behaviour, urine spraying, and hyperactivity were lower following EAD than for cats desexed later in life [60]. However, EAD cats were shyer and more likely to hide [60]. Documented physical effects of EAD included retarded development of the vulva and no development of penile spines [73], neither of which affects health or survival. There is no correlation between the desex age of male cats and urethral diameter [74], or risk of Feline Lower Urinary Tract Disease (FLUTD [75]). Delayed physeal closure occurs in both desexed male and female cats [76,77,78], but with no difference between EAD and TAD [79,80], and no association between EAD and changes in radius length [78] or incidence of long bone fractures [60]. However, desexing and obesity are risk factors for both spontaneous physeal fractures [81] and FLUTD [82]. Desexed animals generally have a greater tendency to gain weight because of reduced metabolic rates [83] and may predispose them to diabetes [84]. However, cat weight is manageable with good husbandry [85,86,87] and should not dissuade owners or veterinary professionals from desexing cats.

Much of this evidence was confirmed in Belgium’s ‘Sterycat Project’—a large-scale initiative of the Belgian government testing the effect of EAD on the health and behaviour of cats [88,89,90,91], as well as offering recommendations and video recordings of surgical techniques and anaesthesia [90]. There was no evidence of increased lower urinary tract disease in either sex, urethral obstruction in males, lameness, fractures, or allergies [91] arising from EAD. Similarly, there was no evidence that EAD contributed to undesirable behaviours in cats followed for up to seven years.

Despite this evidence, concerns regarding health, behaviour and anaesthetic risk persist amongst veterinary professionals in the USA, the UK, New Zealand, and Australia [30,40,44]. Reasons may be complex, including professional judgement and exposure to/interpretation of the available studies [29,30]), lack of practical EAD experience [30], recommendations of national veterinary associations [40], concern about loss of business to private practices if EAD becomes routine before adoption (often compounded by concerns about quality of surgical practice in shelters [30]), and a belief that decisions should be reached in consultation with clients [29,30]. Many of these concerns were present in respondents to our study, but their frequency varied across the professional groups.

### 4.2. Part 1—Preferred Minimum Desexing Ages of Cats and Rationale of Respondents

Although we found no effect of career length on preferred desexing ages, there was disparity in the desexing ages preferred by different professional roles, with later ages preferred by nurses and nursing students. Similar proportions of veterinarians and veterinary students selected a mixture of EAD and TAD for male and female cats (20–30% of both roles preferred 2–3 months, 3–4 months and >4 months). In contrast, more nurses and nursing students preferred TAD (34% and 39% respectively preferred >4 months for males; 44% and 47% for females). Veterinary nurses were also more concerned about anaesthetic risks to female cats, possibly because ovariohysterectomy is invasive and requires general anaesthetic (vs. sedation for castration of males), and because monitoring anaesthesia during surgical procedures is typically the responsibility of nurses. Response weightings of the remaining professional roles revealed that choice of minimum age for male and female cats was most influenced by a desire to reduce the number of stray cats in the community. However, there is a disconnect between attitude and practice, because many respondents concerned about cat overpopulation did not prefer EAD of male cats (28%) or female cats (39%). Therefore, even with good intentions, the age preferences of some professionals and students leave opportunity for female cats to have at least one litter.

### 4.3. Part 2—Online Advice to Pet Owners

Our survey of desexing information available to the public on veterinary practice websites did not reflect many respondent’s wishes to reduce stray and feral populations. Over half of surveyed websites provided no information about desexing cats, or listed desexing as a service, but without explaining why it was necessary, or when to perform it. Given that desexing is a common procedure in small animal practices [92], it is perplexing that many Australian practices exclude relevant information from their websites. Citizens who access the websites of their local veterinarians and find no desexing information may call the practice for more information. However, many people may carry out their own Internet search to help them decide if, and when, to desex their pet. This could lead to misunderstandings of scientific literature or consultation of unreliable sources (e.g., sponsored advertisements, personal blogs).

Nearly 90% of practice websites that specified an age/age range for cats favoured desexing from 4four, five, or six months of age (87% pooled, *n* = 47, 34, 39/138), and is in-line with the personal opinions of veterinarians found in other studies [30,43]. While Farnworth et al. [40] found that Australian veterinarians would desex cats at a minimum mean age of 3.4 months for females and 3.2 months for males, this was not a preference, but the minimum age at which they would consider performing desexing. The AVA is more supportive of EAD than many Australian veterinarians: *“At the veterinary practitioner’s discretion, desexing can be performed from as early as eight weeks of age and at a minimum of 1 kg bodyweight”* [93]. No desexing policy could be found on the VNCA website.

Only two practice websites in the ACT, 1 website in SA and 1 in WA, specifically referred to desexing legislation introduced by their respective governments (3%, *n* = 124). One ACT website openly advertised its willingness to write a medical certificate for pet owners so that local councils would be unable to fine owners for not desexing their pet before the mandated age of three months. Some practices in States with legislation may be unaware or unwilling to promote the legislative responsibilities of cat owners and consequently, the responsibilities of practitioners themselves. This may also explain why some practices do not advertise desexing of cats. Disparity between recommended desexing ages and legislation highlights the need for greater co-operation and communication between legislators and veterinary professionals.

Overall, 97% of practice websites that mentioned desexing for both cats and dogs promoted the same age/age range (*n* = 112/116, 95% recommending TAD). While convenient, this discounts the negative side effects of EAD reported in some dog breeds, in contrast to the extensive evidence of lack of serious effects in cats. Problems for dogs include hip dysplasia, cranial cruciate ligament repair, and sarcomas [37,44,63,94], and research is ongoing. Furthermore, timing of puberty varies with dog sex and breed (e.g., female dogs are generally receptive between four and 12 months, males at 10 months, and small dogs receptive earlier than large dogs [37]). Given the greater variability in response of dogs to EAD than cats, it is logical to recommend different ages for desexing—and comply with both up-to-date literature and any relevant local/State legislation. This includes offering EAD for cats, if not for dogs. In an online research report [28], the RSPCA (Australia) offers detailed factual information on reproductive behaviour of dogs and cats, desexing and recommends desexing cats under 4 months old.

### 4.4. Legislative Context: What’s New in Australia

Surveys in Western Australia and New South Wales indicate consistent support of mandatory desexing of all pet cats other than those owned by licensed breeders by 80% or more of cat owners and non-owners [95,96,97]. This is now reflected in mandatory desexing legislation developed in consultation with the veterinary industry and enacted in the ACT, WA, TAS, and SA [51,52,53,54]. Additionally, some local government municipalities require mandatory desexing. For example, in the State of Victoria 20 local municipalities currently mandate desexing of pet cats [98]; checks of their websites found that 18 require desexing by three months of age, one by six months, and one *“before registration”*. Despite the changing legislative context in Australia, Leung et al. [41] found that government legislation would not influence 82% of veterinarians to desex cats and dogs any younger than their preferred age (*n* = 774). In our study, only two respondents (both veterinarians) specifically stated that their preferred minimum desexing age was guided by law. Twenty-one percent of respondents who chose ‘other’ reasons to justify their preferred age for male and female cats, deferred to private practice protocols and the skill of vets performing surgery.

One veterinary nurse stated that the head veterinarian at her practice was vehemently opposed to desexing under six months and wrote medical certificates for pet owners so that they are not fined by their council for ‘late’ desexing (see Section 3.1.7 of Results). We found a similar offer openly advertised on a practice website in the ACT (where desexing by three months is mandated). The relevant AVA policy states that: *“Veterinarians may recommend desexing of companion animals before puberty unless there is a valid reason to delay the procedure”* [93]. Valid reasons are specific to an individual animal’s medical or welfare concerns, so we do not believe that it should be extended as a general policy by veterinarians or practices.

The AVA encourages surgical desexing in reducing the size of unwanted animal populations [93] but does not currently support mandatory desexing: *“There are inherent problems with the concept of compulsory desexing. Successful enforcement of compliance would depend on universal registration and permanent identification, which have already proven difficult to enforce. Evidence suggests that, at least for cats, compulsory desexing of owned animals would have little effect on the cat population as the majority of over-supply emanates from the semi-owned and unowned populations”* [93]. The views of the VNCA about mandatory desexing of cats is not explicitly stated on the website and is, therefore, unknown.

We agree that enforcement is an essential component of any mandatory legislation but disagree that there is not an opportunity to reduce unwanted cat numbers through increased desexing of owned cats. Marston and Bennett [11] noted that between 2005 and 2006 only 59% of 2,804 owned cats surrendered to a Melbourne shelter were desexed, so at least some cohorts of owners are not desexing their cats. Between 2006 and 2010, national RSPCA shelters reported that 53% of 40,182 owner-surrendered cats were sexually intact. While most adults were desexed (64%, *n* = 21,469), only 27% of kittens were desexed (*n* = 18,713 [10]). Further evidence of low desexing rates for younger cats comes from convenience samples of pet cats presented in 2012 and 2013 (*n* = 584 and 316 respectively) to free microchipping sessions in Western Australia [99]. While 93% of cats over two years old were desexed in 2012 and 97% in 2013, only 49% and 28% of cats under two years old respectively were desexed. Similarly, the Cat Protection Society of Victoria and Animals Australia found that only 37% of pet cats under one year old were desexed (‘The Cat Project’ [27]). Therefore, many cats have the opportunity to breed before being desexed. In a camera survey of incursions by nuisance cats into suburban gardens in Perth, WA, at least 20 of 78 nuisance animals (26%) were identified as sexually intact, and at least 50% of these were identified as owned [100]. There is also evidence of genetic and spatial exchange between the stray and owned cat populations [101]. Alberthsen et al. [10] reported that 24% of 45,099 cats admitted to RSPCA shelters as strays were desexed, so they were either owned at some point or desexed in illegal trap-neuter-return programs. Overall, we believe that these studies show substantial potential for reducing cat populations through enforced mandatory desexing, and EAD.

The current stance on mandatory desexing of cats places the AVA at odds with a substantial majority of the community and with the response to public concerns expressed in cat-specific legislation. There is clearly some way to go in closing the gap between desexing ages recommended by veterinary professionals in Australia and the requirements of at least some state/territory or local government legislation, particularly if more states or local governments implement mandatory desexing legislation.

### 4.5. Exposure of Future Professionals to EAD

Whilst some veterinarians prefer not to operate on small cats, others find surgery on younger animals causes less tissue damage and reduces surgery and recovery times (findings repeatedly asserted by some of our survey respondents and supported by research [30,40]). Preferred ages for desexing decrease as time since graduation increases [40,41], possibly because of experience and increased confidence [30]. Given that only four of the eight universities teaching veterinary science in Australia and New Zealand provide students with opportunities to witness or perform EAD, and that only 24% of veterinarians teaching surgical curricula advocate using EAD (*n* = 6/25 [44]), future student cohorts will benefit from greater exposure to, and experience with, desexing cats under four months, and from opportunities to work alongside surgeons experienced with EAD (e.g., learn gentle surgical dexterity [62]).

Animal shelters are the perfect setting for exposing students to routine EAD. Of the 13 survey respondents in the current study who nominated ‘other’ reasons for selecting male desex ages because of their experience working in shelters or practices that offered shelter-kitten adoption programs, only two were students (one vet student, one nurse student, 0.2%, *n* = 957). No students were amongst the nine respondents who nominated ‘other’ desex ages for female cats because of their experience with shelters/adoption programs. It, therefore, appears there is a gap between students gaining ‘real world’ experience with EAD and the ample opportunities to practice EAD that shelters could provide. Practical placements would provide opportunity to witness and perform multiple desexing surgeries at regular intervals (and other services, e.g., diagnosing and treating FIV), improve animal handling, become familiar with the scale of unwanted cats in the local community, and appreciate the limited resources at most shelters [102,103,104,105,106,107,108]. Shelters would also benefit by gaining free workers that assist in many tasks (e.g., rehabilitation, enrichment, training, educating the public, euthanasia, quarantine, cleaning). 

In 2017, veterinary students from Australia and New Zealand identified desexing, companion animal husbandry, and euthanasia as the top three welfare and ethics issues they most needed competency in before work placement [109]. Employers of graduates reported that graduates needed greater elective surgery skills [92] and improved interpersonal communication skills with clients and nurses [110,111]. Approximately 50% of veterinary students surveyed internationally stated that their institutions did not provide enough training of practical skills (e.g., reproduction, surgery), and up to 35% of students did not feel they had been taught adequate socio-economic skills (e.g., communication, management [112]). Students placed at shelters would have opportunity to improve proficiency in these areas and be empowered to make educated, ethical decisions about desexing and euthanasia in their future career [113,114]. Veterinary science and nursing student curricula could use cat case studies to introduce students to social and ecological implications of large pet and unwanted cat populations (e.g., euthanasia of healthy animals, disease [3,115]).

### 4.6. Exposure of Current Professionals to EAD

Leung et al. [41] found that 45% of Australian and New Zealand veterinarians would consider lowering their recommended desexing age if they felt there was adequate support in the literature; an additional 10% would lower recommended ages if there was government legislation; public demand for lower desexing ages (10%), mentor/academic approval of lower desexing ages (9%), peer approval (5%), and if technique workshops were offered (3%). Internationally, veterinarians remain unsure about the risks and benefits of EAD or believe the subject has not been researched thoroughly [30]. Murray et al. [43] found that UK veterinarians were less likely to believe EAD was appropriate for cats if they believed surgical, anaesthetic and urogenital complications would be higher than if cats were desexed at six months (TAD). The attitudes of veterinary nurses are important too, because in several countries they can provide independent client consultations that include advice about desexing [116,117]. Veterinarians and nurses who are currently practicing also need to be empowered with knowledge of cutting-edge cat research. This is already achievable if vets and nurses join the International Society of Feline Medicine [118] which provides role-specific webinars, newsletters, and forums that address cat-specific topics, as well as access to the latest science in the Journal of Feline Medicine and Surgery and/or Feline Focus. Another initiative is the Kitten Neutering Database or ‘KiND’ [26,119]. Established in the UK, approximately 1400 veterinary practices have registered with KiND. It provides a public search tool enabling owners to find a vet who will neuter their cat at four months of age. In addition to providing this resource for owners, the aims of KiND are to:

- “Work to ensure all veterinary practices promote and practise neutering at four months (the age at which cats can get pregnant)
- Ensure all rescue organisations should adopt policies to neuter prior to rehoming
- Re-focus neutering education campaigns to ensure they reposition neutering as the act of a caring, loving cat owner
- Ensure the ‘one litter myth’ is dispelled
- Encourage collaboration between animal welfare and rescue organisations, the veterinary profession and housing associations through community outreach programmes to target those audiences less likely to neuter
- Encourage pro-bono support by veterinary professionals to further animal welfare”.

To achieve this, KiND offers educational materials for owners and veterinary professionals (e.g., anaesthetic protocols and training videos that showcase EAD). It is a support network for professionals and could prove a useful model in Australia and elsewhere. Industry representative bodies could also consider providing courses developed in collaboration with local governments to update professionals with changing animal legislation and the legal responsibilities of individuals and businesses.

### 4.7. Other Barriers to Uptake of EAD

Frank and Carlisle-Frank [120] examined data from private veterinary practices and shelters offering discounted desexing across large counties in five states of the USA. They found that implementing low-cost desexing programs did not reduce the number of desexing procedures performed in private practices for regular fees. Despite this, Heath [121] posits that competition between veterinarians for business will increase if numbers of dogs and cats decrease over time. In our study, one survey respondent (a vet) claimed that mandatory desexing is bad for business as more breeding pet cats meant more income. Given that pet cat ownership in Australia is stable (approximately 3.3 million cats/2.3 million households in 2013 (1.43 cats/household [122]) vs. 3.9 million cats/2.7 million households in 2016 (1.44 cats/household [22]), it may be prudent for practices to consider partnering with shelters to guarantee business.

The number of vets and vet nurses currently providing services to Australian shelters is unknown. However, nearly half of Australian graduate veterinarians enter small animal practices (48% [23]), and the same situation may apply to nurses (66% of UK nurses work in small animal practice [117]). Many professionals remain in private practice for the duration of their career (85% of veterinarians are still in private practice after ≥15 years [123]) and may consequently have little exposure to shelters and the challenges they face. If both professions rarely work with shelters or participate in EAD, they are unlikely to see a need for the practice even if the number of cats needing desexing and rehoming increases (e.g., some vets recommend cats have a season/litter before desexing [41], and one surveyed nurse believes professionals practicing EAD should be prosecuted for cruelty). Many practices overseas already offer the public discounted desexing services [120] and, in Australia, practices can partner with organisations such as the National Desexing Network or ‘NDN’ [124] to offer disadvantaged pet owners cheaper desexing, but membership is low. Research and statistical modelling that could account for changes to revenue from shelter partnering would be of value, as would future projections of shelter and pet populations if more desexing was achieved.

### 4.8. Study Limitations

Data presented are for minimum ages that suit the personal opinions and experience of the respondents. Data do not reflect the ages at which cats are most frequently desexed in private practices or at shelters. This data would provide valuable insight into the topic of desexing in Australia.

This survey of attitudes towards minimum desexing ages is essentially a study of women. Females are often overrepresented in self-selected surveys [125], however, the veterinary profession is increasingly dominated by females in Australia and overseas [111,126,127,128,129,130,131]. This change in demographic could retard efforts to reduce unwanted cat populations as female vets are less likely to recommend EAD for cats than male vets [40]. Female professionals are also generally more empathetic towards animals [132,133] and experience greater discomfort with euthanasia-for-convenience scenarios than males [134]. Euthanasia of unwanted cats is likely to cause stress and burnout in some professionals [135,136]. Additionally, many female vets and nurses exit the profession to have children before returning part-time [110,117,126,127,128,129,131]. Veterinarian age and career length are negatively associated with preferred desexing age [40,41], therefore, female vets and nurses who cycle between practicing and non-practicing risk falling out of synchronicity with the latest scientific research on cats and may consequently favour conservative desexing ages. More male-centric research into preferred minimum desexing ages for cats would be valuable in determining whether the trend towards conservative ages is industry-wide.

## 5. Conclusions

Veterinarians, veterinary nurses, and students of both disciplines, play a crucial role in educating the public who apply to them for advice and services that optimise animal health and welfare—in person, over the phone or via websites. Their professional opinions and preferences for if, and when, cats should be desexed have significant influence. Their knowledge should, therefore, reflect up-to-date developments and findings in scientific literature and comply with animal legislation.

Professionals in Australian private practices may not yet euthanize large numbers of healthy animals; however, resistance to EAD for pet cats may none-the-less contribute to shelter overload. Whilst managing unowned cat populations challenges authorities and requires further research and legislative enforcement, initiatives from the whole veterinary industry can achieve consistent, high desexing rates of pet cats across Australia. Evidence clearly favours the safety and practicability of EAD for cats, so greater implementation of EAD could do no harm and might reduce the number of accidental and unwanted cats surrendered to shelters annually.

## Figures and Tables

**Table 1:** Classification of respondents to the face-to-face and online surveys. Forty-four respondents specified two professional roles, so their responses were counted once per role. The surveys of 16 non-professionals and 10 respondents with no profession were excluded from statistical analyses. An additional 47 respondents logged-on and then logged-off without completing the survey and were excluded from analyses.

Survey Method	Professional Role	Respondents *n*	Excluded *n*	+ 2x Roles *n*	Analysed *n*
2015 VNCA Conference Face-to-face Surveys	Vets	1	0	0	1
	Nurses	133	0	3	136
	Vet Students	0	0	0	0
	Nurse Students	2	0	1	3
	Others	1	0	4	5
	Total *n*	137	0	8	145
2014 AVA Conference Face-to-face Surveys	Vets	20	0	2	22
	Nurses	12	0	5	17
	Vet Students	17	0	2	19
	Nurse Students	0	0	0	0
	Others	14	13	1	2
	Total *n*	63	13	10	60
Online Surveys SurveyMonkey© Link	Vets	35	0	3	38
	Nurses	139	0	31	170
	Vet Students	252	0	25	277
	Nurse Students	250	0	1	251
	Others	9	3	10	16
	No profession stated	10	10	0	0
	Logged-off	47	47	0	0
	Total *n*	742	60	70	752
	Grand Total *n*	942	73	88	957

AVA: Australian Veterinary Association. VNCA: Veterinary Nurses Council of Australia.

**Table 2:** Career length of 957 veterinary professionals and students replying to face-to-face and online surveys about preferred minimum desexing ages for male and female cats.

Professional Role	Vets		Nurses		Vet Students		Nurse Students		Others		Total	
	*n*	%	*n*	%	*n*	%	*n*	%	*n*	%	*n*	%
Career Length												
<1 year	3	4.9	9	2.8	248	83.8	140	55.1	1	4.3	401	41.9
1‒5 years	10	16.4	117	36.2	42	14.2	106	41.7	8	34.8	283	29.6
6‒10 years	5	8.2	86	26.6	3	1.0	3	1.2	7	30.4	104	10.9
11‒15 years	5	8.2	46	14.2	0	0.0	0	0.0	2	8.7	53	5.5
16‒20 years	9	14.8	36	11.1	2	0.7	2	0.8	1	4.3	50	5.2
>21 years	28	45.9	26	8.0	1	0.3	3	1.2	4	17.4	62	6.5
No answer	1	1.6	3	0.9	0	0.0	0	0.0	0	0.0	4	0.4
Total *n*	61	100.0	323	99.8	296	100.0	254	100.0	23	99.9	957	100.0

**Table 3:** Preferred minimum desexing ages for male and female cats selected by 957 professionals and students within the veterinary industry.

***Male Cats***												
**Professional Role**	**Vets**		**Nurses**		**Vet Students**		**Nurse Students**		**Other**		**Total**	
	***n***	**%**	***n***	**%**	***n***	**%**	***n***	**%**	***n***	**%**	***n***	**%**
**Desex Ages**												
<1 month	0	0.0	1	0.3	3	1.0	2	0.8	0	0.0	6	0.6
1‒2 months	7	11.5	13	4.0	38	12.8	22	8.7	0	0.0	80	8.4
2‒3 months	13	21.3	62	19.2	59	19.9	42	16.5	6	26.1	182	19.0
3‒4 months	17	27.9	73	22.6	57	19.2	57	22.4	7	30.4	211	22.0
>4 months	15	24.6	110	34.1	53	17.9	98	38.6	6	26.1	282	29.5
Other age	5	8.2	51	15.8	5	1.7	7	2.8	1	4.3	69	7.2
No answer	4	6.5	13	4.0	81	27.4	26	10.2	3	13.0	127	13.3
Total *n*	61	100.0	323	100.0	296	99.9	254	100.0	23	99.9	957	100.0
***Female Cats***												
**Professional Role**	**Vets**		**Nurses**		**Vet Students**		**Nurse Students**		**Other**		**Total**	
	***n***	**%**	***n***	**%**	***n***	**%**	***n***	**%**	***n***	**%**	***n***	**%**
**Desex Ages**												
<1 month	0	0.0	1	0.3	0	0.0	1	0.4	0	0.0	2	0.2
1‒2 months	4	6.6	5	1.5	25	8.4	16	6.3	0	0.0	50	5.2
2‒3 months	14	23.0	43	13.3	42	14.2	30	11.8	3	13.0	132	13.8
3‒4 months	18	29.5	70	21.7	62	20.9	50	19.7	11	47.8	211	22.0
>4 months	16	26.2	142	44.0	73	24.7	120	47.2	5	21.7	356	37.2
Other age	5	8.2	46	14.2	6	2.0	7	2.8	1	4.3	65	6.8
No answer	4	6.5	16	5.0	88	29.7	30	11.8	3	13.0	141	14.7
Total *n*	61	100.0	323	100.0	296	99.9	254	100.0	23	99.8	957	99.9

**Table 4:** ‘Other’ category minimum desexing ages for male and female cats nominated by 69 and 65 survey respondents respectively (7% of *n* = 957). Respondents mostly provided *“if/or”* answers (55%, *n* = 38 for male cats; 60%, *n* = 39 for female cats).

***Male Cats***							
**Professional Role**	**Vets**	**Nurses**	**Vet Students**	**Nurse Students**	**Other**	**Total *n***	**%**
**Other Desex Ages**							
Before problem behaviour **^1^**	0	2	0	1	0	3	4.3
Developmental age **^2^**	0	1	0	0	0	1	1.4
Body weight **^3^**	3	13	2	2	1	21	30.4
*IF* Age *OR* Body weight **^4^**	1	10	1	1	0	13	18.8
*IF* Age *OR* Development **^5^**	0	4	0	0	0	4	5.8
*IF* Body weight *OR* Development **^6^**	0	2	0	0	0	2	2.9
*IF* Private practice *OR* Shelter **^7^**	1	17	0	1	0	19	27.5
Alternatives **^8^**	0	2	2	2	0	6	8.7
Total *n*	5	51	5	7	1	69	99.8
%	7.2	73.9	7.2	10.1	1.4	99.8	
***Female Cats***							
**Professional Role**	**Vets**	**Nurses**	**Vet Students**	**Nurse Students**	**Other**	**Total *n***	**%**
**Other Desex Ages**							
Before problem behaviour **^1^**	0	0	0	0	0	0	0.0
Developmental age **^2^**	0	1	0	1	0	2	3.1
Body weight **^3^**	3	11	1	3	1	19	29.2
*IF* Age *OR* Body weight **^4^**	1	10	1	1	0	13	20.0
*IF* Age *OR* Development **^5^**	0	3	1	0	0	4	6.2
*IF* Body weight *OR* Development **^6^**	0	0	0	0	0	0	0.0
*IF* Private practice *OR* Shelter **^7^**	1	21	0	0	0	22	33.8
Alternatives **^8^**	0	0	3	2	0	5	7.7
Total *n*	5	46	6	7	1	65	100.0
%	7.7	70.8	9.2	10.8	1.5	100.0	

**^1.^** Before problem behaviour included: *“Desex before urine spraying/fighting/roaming starts”*. **^2.^** Developmental age included: *“Desex after testes have descended; before/after puberty”*. **^3.^** Minimum body weight nominated: *“Desex from 800 g/1.0 kg/1.2 kg/1.5 kg/2.0 kg”*. **^4.^** IF Age OR Body weight: e.g., *“Desex cats IF ≥4 months OR after 1.0 kg—whichever comes first”*. **^5.^** IF Age OR Development: e.g., *“Desex cats IF ≥4 months OR before first heat—whichever comes first”*. **^6.^** IF Body weight OR Development: e.g., *“Desex cats IF weighing ≥1.0 kg OR as soon as testes are descended—whichever comes first”*. **^7.^** IF Private practice OR Shelter: e.g., *“Desex cats at 6 months IF presented to private practices OR from 1.0 kg if cats are in shelters”*. **^8.^** Alternative ages included: e.g., *“Desex at any age; Assessed on a case-by-case basis; Age determined only by owner; Not taught an age at school yet; Have not thought about it”*.

**Table 5:** Weighted frequencies of reasons chosen by 831 veterinary professionals and students to justify their choice of minimum desexing ages for male and female cats (answer equals approximately 1.00 per person, answers to two decimal places).

***Male Cats***							
**Professional Role**	**Vets**	**Nurses**	**Vet Students**	**Nurse Students**	**Other**	**Total Response Weight**	**%**
**Desex Reasons**							
A. Anaesthetic risk	13.41	77.15	46.58	68.33	4.49	209.96	25.3
B. Lack of experience	6.57	16.31	39.45	16.38	0.00	78.71	9.5
C. Abnormal development	8.07	71.00	56.68	57.01	4.49	197.25	23.8
D. Reduce no. strays	19.41	83.01	64.51	78.16	8.16	253.25	30.5
E. Other	9.50	62.21	7.48	8.83	2.83	90.85	10.9
Total Response Weight	56.96	309.68	214.70	228.71	19.97	830.02	100.0
***Female Cats***							
**Professional Role**	**Vets**	**Nurses**	**Vet Students**	**Nurse Students**	**Other**	**Total Response Weight**	**%**
**Desex Reasons**							
A. Anaesthetic risk	12.91	87.21	45.05	67.15	3.66	215.98	26.6
B. Lack of experience	7.07	16.63	43.16	14.89	0.00	81.75	10.1
C. Abnormal development	8.07	58.24	50.64	59.51	4.66	181.12	22.3
D. Reduce no. strays	16.41	81.73	60.30	77.99	7.83	244.26	30.0
E. Other	12.50	62.80	6.40	4.16	3.83	89.69	11.0
Total Response Weight	56.96	306.61	205.55	223.70	19.98	812.80	100.0

**Table 6:** Summary of ‘Other’ reasons for choice of minimum desexing ages for male and female cats were provided by 140 and 130 respondents respectively (15% and 14% of *n* = 957). Respondents could provide ≥1 reason.

***Male Cats***							
**Professional Role**	**Vets**	**Nurses**	**Vet Students**	**Nurse Students**	**Other**	**Total**	**%**
**Respondents *n***	**15**	**92**	**15**	**14**	**4**	**140**	
**Other Reasons for Chosen Desex Age**							
1. Before problem behaviour/sexual maturity	1	28	5	5	1	43	24.9
2. Practice protocol or based on surgeon skill/veterinary preferences	7	28	2	3	2	36	20.8
3. Easier/safer surgery	2	16	2	3	2	30	17.3
4. Desexes for shelter or has practice adoption program	0	9	1	1	0	13	7.5
5. Work in low income area/Cannot rely on owner’s motivation	0	9	0	0	0	9	5.2
6. Based on wishes of owner/breeder	2	5	1	2	0	8	4.6
7. Easier to rehome/cost-effective surgery at age	0	3	0	0	0	5	2.9
8. Positive experiences with younger ages	0	5	0	0	0	5	2.9
9. Negative experiences with younger ages	0	1	1	0	0	2	1.2
10. Emotional decision/Welfare concerns	0	2	2	0	0	4	2.3
11. Tradition	0	2	0	0	0	2	1.2
12. Law	2	0	0	0	0	2	1.2
13. AVA recommendation	0	0	1	0	0	1	0.6
14. Unsure/Not thought about it	0	0	2	0	0	2	1.2
15. Additional reasons *	2	6	1	2	0	11	6.4
Total Reasons *n*	20	114	18	16	5	173	100.2
***Female Cats***							
**Professional Role**	**Vets**	**Nurses**	**Vet Students**	**Nurse Students**	**Other**	**Total**	**%**
**Respondents *n***	**16**	**90**	**12**	**7**	**5**	**130**	
**Other Reasons for Chosen Desex Age**							
1. Before problem behaviour/sexual maturity	4	19	3	1	1	27	16.9
2. Practice protocol or based on surgeon skill/veterinary preferences	1	29	1	1	2	34	21.3
3. Easier/safer surgery	7	19	1	3	3	33	20.6
4. Desexes for shelter or has practice adoption program	2	7	0	0	0	9	5.6
5. Work in low income area/Cannot rely on owner’s motivation	1	10	0	0	0	11	6.9
6. Based on wishes of owner/breeder	0	6	0	0	0	6	3.7
7. Easier to rehome/cost-effective surgery at age	2	3	0	0	0	5	3.1
8. Positive experiences with younger ages	0	5	0	0	0	5	3.1
9. Negative experiences with younger ages	1	4	1	0	0	6	3.7
10. Emotional decision/Welfare concerns	0	1	2	0	0	3	1.9
11. Tradition	1	2	0	0	0	3	1.9
12. Law	2	0	0	0	0	2	1.3
13. AVA recommendation	0	0	1	0	0	1	0.6
14. Unsure/Not thought about it	0	0	1	0	0	1	0.6
15. Additional reasons *	3	7	2	2	0	14	8.8
Total Reasons *n*	24	112	12	7	6	160	100.0

AVA: Australian Veterinary Association. * Additional reasons included: *“There are no advantages to EAD (Early Age Desexing)”*, *“Cats need to be fully vaccinated before desexing”*, *“There is no registered anaesthetic for neonatal kittens”*, *“Reduces cancer risk”, “Not taught at school yet”*, and *“I would use the literature”*.

**Table 7:** Frequency of reasons chosen by veterinary professionals and students to justify their choice of preferred minimum desexing age for male and female cats.

***Male Cats***							
**Professional Role**	**Vets**	**Nurses**	**Vet Students**	**Nurse Students**	**Other**	**Total *n***	**%**
**Number of Reasons**							
0	4	13	81	25	3	126	13.2
1	32	165	85	117	13	412	43.0
>1	25	145	130	112	7	419	43.8
Total *n*	61	323	296	254	23	957	100.0
***Female Cats***							
**Professional Role**	**Vets**	**Nurses**	**Vet Students**	**Nurse Students**	**Other**	**Total *n***	**%**
**Number of Reasons**							
0	4	16	90	30	3	143	14.9
1	38	157	77	109	13	394	41.2
>1	19	150	129	115	7	420	43.9
Total *n*	61	323	296	254	23	957	100.0

**Table 8:** Number and percentage of survey respondents (unweighted responses) selecting specific reasons to justify their choice of preferred minimum desexing age for male and female cats.

***Male Cats***										
**Desex Age Category**	**EAD**		**TAD and MAD**		**Other**		**No answer**		**Total Professionals**	
	***n***	**%**	***n***	**%**	***n***	**%**	***n***	**%**	***n***	**%**
**Desex Reasons**										
A. Anaesthetic risk	185	50.4	166	45.2	14	3.8	2	0.5	367	99.9
B. Lack of experience	99	66.9	45	30.4	3	2.0	1	0.7	148	100.0
C. Abnormal development	172	51.7	152	45.6	8	2.4	1	0.3	333	100.0
D. Reduce no. strays	404	78.3	72	13.9	40	7.8	0	0	516	100.0
E. Other	59	42.1	46	32.9	33	23.6	2	1.4	140	100.0
***Female Cats***										
**Desex Age Category**	**EAD**		**TAD and MAD**		**Other**		**No Answer**		**Total Professionals**	
	***n***	**%**	***n***	**%**	***n***	**%**	***n***	**%**	***n***	**%**
**Desex Reasons**										
A. Anaesthetic risk	156	40.9	208	54.6	16	4.2	1	0.3	381	100.0
B. Lack of experience	85	54.8	69	44.5	1	0.6	0	0.0	155	99.9
C. Abnormal development	140	43.2	177	54.6	7	2.2	0	0.0	324	100.0
D. Reduce no. strays	249	61.3	120	29.6	37	9.1	0	0.0	406	100.0
E. Other	42	32.3	55	42.3	33	25.4	0	0.0	130	100.0

EAD: Early Age Desexing. TAD: Traditional Age Desexing. MAD: Mature Age Desexing.

**Table 9:** Results of three-way log-linear analyses assessing the influence of professional role/career length and cat sex on choice of preferred minimum desexing ages (Appendix D, Table A4, Table A5, Table A6, Table A7 and Table A8).

Questions about Minimum Desex Ages	Significant Interactions	G**^2^**	d.f.	*p* Value	Interpretation	Data Tables in Appendix D
Do professionals and students prefer the same min. desex ages for male and female cats?	Profession vs. Cat Sex vs. Desex Age	203.60	40	<0.0001 *	Professional role influenced preferred min. desex age for male and female cats. Nurses and nursing students were more conservative than vets or vet students, preferring to desex both sexes >4 months.	D1
Does vet career length influence preferred min. desex ages for male and female cats?	Career Length vs. Cat Sex vs. Desex Age	10.20	22	0.98	Vet career length did not influence preferred min. desex ages for male and female cats.	D2
Does nurse career length influence preferred min. desex ages for male and female cats?	Career Length vs. Cat Sex vs. Desex Age	27.40	22	0.20	Nurse career length did not influence preferred min. desex ages for male and female cats.	D3
	Desex Age vs. Cat Sex	11.22	4	0.02	However, cat sex did influence min. desex age with nurses preferring to desex female cats at older ages than males.	
Does vet student career length influence preferred min. desex ages for male and female cats?	Career Length vs. Cat Sex vs. Desex Age	15.78	13	0.26	Vet student career length did not influence preferred min. desex ages for male and female cats.	D4
	Desex Age vs. Cat Sex	10.16	4	0.04	However, cat sex did influence min. desex age with vet students preferring to desex female cats at older ages than males.	
Does nurse student career length influence preferred min. desex ages for male and female cats?	Career Length vs. Cat Sex vs. Desex Age	16.08	13	0.25	Nurse student career length did not influence preferred min. desex ages for male and female cats.	D5

d.f.: degrees of freedom. * *p* Values significant after Bonferroni correction.

**Table 10:** Results of three-way log-linear analyses assessing the influence of professional role/career length and cat sex on the reasons chosen to justify preferred minimum desexing ages (Appendix E, Table A9, Table A10, Table A11, Table A12 and Table A13).

*Questions about Minimum Desex Age Reasons*	Significant Interactions	G**^2^**	d.f.	*p* Value	Interpretation	Data Tables in Appendix E
Do professionals and students prefer min. desex ages of male or female cats for the same reasons?	Professional Role vs. Cat Sex vs. Desex Reason	201.64	40	<0.0001 *	Professional role influenced reasons chosen to justify preferred min. desex ages of male or female cats. Across roles, the main reason for desexing males at a specific age was to reduce stray populations. For female cats, vets, vet students and nursing students also wanted to reduce strays. Nurses were more motivated by anaesthetic risk to females.	E1
Does vet career length influence reasons for preferred min. desex ages for male or female cats?	Career Length vs. Cat Sex vs. Desex Reason	20.36	22	0.56	Vet career length did not influence reasons chosen to justify preferred min. desex ages for male or female cats.	E2
Does nurse career length influence reasons for preferred min. desex ages for male or female cats?	Career Length vs. Cat Sex vs. Desex Reason	18.64	22	0.67	Nurse career length did not influence reasons chosen to justify preferred min. desex ages for male or female cats.	E3
	Career Length vs. Desex Reason	16.66	8	0.03	However, career length did influence desex reason. Nurses with ≤5 yrs experience were most motivated in choice of desex age by anaesthetic risk. Nurses with >5 yrs experience were more motivated to reduce stray populations.	
Does vet student career length influence reasons for preferred min. desex ages for male or female cats?	Career Length vs. Cat Sex vs. Desex Reason	6.20	13	0.94	Vet student career length did not influence reasons chosen to justify preferred min. desex ages for male or female cats.	E4
Does nurse student career length influence reasons for preferred min. desex ages for male or female cats?	Career Length vs. Cat Sex vs. Desex Reason	4.64	13	0.98	Nurse student career length did not influence reasons chosen to justify preferred min. desex ages for male or female cats.	E5

d.f.: degrees of freedom. * *p* Values significant after Bonferroni correction.

**Table 11:** Desktop survey of internet websites belonging to 299 urban veterinary practices in the capital cities of six states and two territories of Australia. Initial survey of four states was carried out in 2016 and extended to include other states and territories in 2018.

State		NSW	VIC	QLD	WA **^1.^**	SA **^2.^**	TAS **^3.^**	ACT **^4.^**	NT		
Year of Survey		2016	2016	2016	2016	2018	2018	2018	2018		
Legislation Years *		0	0	0	4	1	10	5	0		
										Total	%
Websites *n*		50	50	50	50	50	15	24	10	299	100
***Cats***	**Responses**										
**Q1.**	Yes	23	24	24	22	23	5	6	8	135	45.2
*n* = 299	No	27	26	26	28	27	10	18	2	164	54.8
**Q2.**	Yes	23	20	18	22	23	5	6	8	125	92.6
*n* = 135	No	0	4	6	0	0	0	0	0	10	7.4
***Dogs***											
**Q1.**	Yes	23	27	26	22	24	5	6	8	141	47.2
*n* = 299	No	27	23	24	28	26	10	18	2	158	52.8
**Q2.**	Yes	23	21	24	22	24	5	5	8	132	93.6
*n* = 141	No	0	6	2	0	0	0	1	0	9	6.4
***Cats and Dogs***											
**Q3.**	Yes	22	23	17	20	21	4	3	6	116	87.2
*n* = 133	No	1	1	7	2	1	1	2	2	17	12.8

**^1.^** From 2013, Western Australia (WA) enforced legislation that all pet cats are to be desexed by six months of age. **^2.^** From 2018, South Australia (SA) will enforce legislation that all pet cats are to be desexed by six months of age. **^3.^** From 2009, Tasmania (TAS) enforced legislation that all pet cats are to be desexed by six months of age. **^4.^** In 2014, the Australian Capital Territory (ACT) altered legislation (from 2000) mandating that cats be desexed by three months of age. * Number of years that legislation had been in place in states and territories during the years in which desktop surveys were conducted. NT: Northern Territory. QLD: Queensland. VIC: Victoria. NSW: New South Wales. Q1. Did the website specify a desexing age/age range? Q2. Was the specified desexing age/age range the same for both sexes? Q3. Of the websites that provided a desexing age/age range for both cats and dogs, were the ages the same?

**Table 12:** Nearly all Australian veterinary practice websites that provided information about when to desex cats or dogs, recommended the same age/age range for both males and females (93% cats vs. 94% dogs). Fewer websites recommended desexing males and females at different ages (10 for cats vs. nine for dogs, three websites provided ages for female cats and dogs only). When the age/range listed by websites were grouped into desex age categories * or ‘general statements’ about timing of desexing, it was evident that practices predominantly recommend desexing cats or dogs at ages classified as TAD.

	*Cats*			*Dogs*		
Desex Age/Range Listed	Same Age	Different Age		Same Age	Different Age	
	Both Sexes	Males	Females	Both Sexes	Males	Females
**Total Websites *n***	125	7	10	132	4	9
**% of Total Websites with Age/Range provided** **(*n* = 135 cats, *n* = 141 dogs)**	92.6	5.2	7.4	93.6	2.8	6.4
Desex Age Category * = EAD						
From 2 months	3	0	0	1	0	0
From 3 months	1	0	1	1	1	0
Before 3 months	2	0	0	0	0	0
Total *n*	6	0	1	2	1	0
%	4.8	0.0	10.0	1.5	25.0	0.0
Desex Age Category * = TAD						
From 4 months	46	1	0	48	1	0
From 5 months	29	1	5	27	0	2
From 6 months	35	4	0	45	1	2
Before 6 months	4	0	0	5	1	0
Total *n*	114	6	5	125	3	4
%	91.2	85.7	50.0	94.7	75.0	44.4
Desex Age Category * = MAD						
Before 12 months	1	0	0	1	0	0
Before 24 months	2	0	2	2	0	2
Total *n*	3	0	2	3	0	2
%	2.4	0.0	20.0	2.3	0.0	22.2
**General Statements**						
Pre-puberty **^1.^**	1	1	2	1	0	3
Varies by weight/breed **^2.^**	0	0	0	1	0	0
≥1.2 kg **^3.^**	1	0	0	0	0	0
Total *n*	2	1	2	2	0	3
%	1.6	14.3	20.0	1.5	0.0	33.3

* Desex category defined as: EAD < 4 months; TAD 4–6 months; and MAD > 6 months. ****^1.^**** Relies on correct identification of puberty by owner and that owners can anticipate the event in cats and dogs. ****^2.^**** Relies on owner knowing the specific requirements for desexing specific breeds of cats and dogs. ****^3.^**** Relies on owner knowing body weight of pet cat or dog.

**Table 13:** Nearly 97% of Australian veterinary practice websites that specified a desexing age for both cats and dogs, recommended the same age/age range for both species (*n* = 112/116). Fewer websites recommended desexing males and females at different ages (one for cats vs. four for dogs, the websites provided ages for female animals only). Most websites recommended TAD for both species.

	*Cats AND Dogs*		
Desex Age/Range Listed	Same Age	Different Age	
	Both Sexes	Males	Females
**Total Websites *n***	112	1	4
**% of Total Websites with Age/Range provided** **(both species *n* = 116)**	96.6	0.9	3.4
Desex Age Category * = EAD			
From 2 months	1	0	0
From 3 months	1	0	0
Total *n*	2	0	0
%	1.8	0.0	0.0
Desex Age Category * = TAD			
From 4 months	46	1	0
From 5 months	24	0	1
From 6 months	34	0	0
Before 6 months	2	0	0
Total *n*	106	1	1
%	94.6	100.0	25.0
Desex Age Category * = MAD			
Before 12 months	1	0	0
Before 24 months	2	0	2
Total *n*	3	0	2
%	2.7	0.0	50.0
**General Statements**			
Pre-puberty **^1.^**	1	0	1
Total *n*	1	0	1
%	0.9	0.0	25.0

* Desex category defined as: EAD < 4 months; TAD 4–6 months; and MAD > 6 months. **^1.^** Relies on correct identification of puberty by owner and that owners can anticipate the event in cats and dogs.

## Appendix A

**Table A1:** Format of face-to-face and online surveys given to professionals and students in the veterinary industry to assess preferences and rationale for minimum desex ages for male and female cats.

**Section 1: Demographic Information**	
Are you:	Male
	Female
Are you a member of:	Australian Veterinary Association
	Veterinary Nurses Council of Australia
	Both
	Neither
Are you a:	Veterinarian
	Veterinary nurse
	Veterinary student
	Veterinary nurse student
	Other
How many years have you been in practice?	<1 year
	1–5 years
	6–10 years
	11–15 years
	16–20 years
	>21 years
	Other
What is the postcode of your current or previous practice?	
**Section 2: Male Desexing Age and Rationale**	
What is the youngest age at which you would prefer to desex MALE cats?	≤ 1 month (≤ 4 weeks)
	2 months (5–8 weeks)
	3 months (9–12 weeks)
	4 months (13–16 weeks)
	>4 months (>17 weeks)
	Other
For what reason/s do you choose to desex MALE cats at the specified age?	A. You believe there is less anaesthetic risk at the selected age
	B. You have limited experience with desexing cats younger than the selected age
	C. You have concerns about normal development of anatomy/physiology/behaviour in cats younger than the selected age
	D. Desexing at the selected age will help reduce the number of stray/abandoned/feral cats in the community
	E. Other
**Section 3: Female Desexing Age and Rationale**	
What is the youngest age at which you would prefer to desex FEMALE cats?	≤ 1 month (≤ 4 weeks)
	2 months (5–8 weeks)
	3 months (9–12 weeks)
	4 months (13–16 weeks)
	>4 months (>17 weeks)
	Other
For what reason/s do you choose to desex FEMALE cats at the specified age?	A. You believe there is less anaesthetic risk at the selected age
	B. You have limited experience with desexing cats younger than the selected age
	C. You have concerns about normal development of anatomy/physiology/behaviour in cats younger than the selected age
	D. Desexing at the selected age will help reduce the number of stray/abandoned/feral cats in the community
	E. Other

## Appendix B

**Table A2:** The initial 2016 desktop survey of veterinary practices in New South Wales revealed that within 50 km of the Sydney CBD there were five chains of private practices and two shelter groups with associated veterinary practices. Within each chain (*n* = 38) and shelter group (*n* = 8), practices recommended the same ages/no age for cats and/or dogs. Including desexing data from these chains and shelter groups in survey analyses would over-represent the recommended ages.

			*Cats*			*Dogs*	
Practice Type	Practices *n*	Same Age Recommended by All Practices	Same Age Both Sexes	Age (months)	Same Age Cats and Dogs	Same Age Both Sexes	Age (months)
Chain 1	13	Yes	Yes	5–6	No	Yes	6
Chain 2	4	Yes	Yes	*****	No	Yes	******
Chain 3	13	ND	ND	ND	ND	ND	ND
Chain 4	3	Yes	Yes	4–6	Yes	Yes	4–6
Chain 5	5	ND	ND	ND	ND	ND	ND
Shelter Group 1	5	Yes	Yes	From 2	Yes	Yes	From 2
Shelter Group 2	3	Yes	Yes	From 4	Yes	Yes	From 4

***** Refers client to a website offering veterinary advice that suggests cats should be desexed *“before sexual maturity”*. ****** Refers client to a website offering veterinary advice that suggests dogs should be desexed after six months because of emerging evidence against traditional age desexing (TAD). ND: No Data available.

## Appendix C

**Table A3:** Preferred minimum desex age for male and female cats were compared between survey modes for each professional role. The number of vet students, nursing students and ‘other’ professionals who completed face-to-face surveys were too small for valid comparisons with students who completed online surveys—therefore, no χ^2^ analysis were run for these cohorts. ‘Other’ desex ages were excluded from analyses as these responses were explored and described independently from pre-determined age categories.

***Male Cats***										
**Professional Role**	**Veterinarians**		**Veterinary Nurses**		**Vet Students**		**Nurse Students**		**Other**	
**Survey Type**	**A**	**B**	**A**	**B**	**A**	**B**	**A**	**B**	**A**	**B**
**Desex Age**										
<1 month	0	0	1	0	0	3	0	2	0	0
1–2 months	1	6	2	11	2	36	0	22	0	0
2–3 months	8	5	20	42	5	54	0	42	1	5
3–4 months	4	13	36	37	3	54	0	57	3	4
>4 months	7	8	52	58	8	45	2	96	2	4
Total *n*	20	32	111	148	18	192	2	219	6	13
***Female Cats***										
**Professional Role**	**Veterinarians**		**Veterinary Nurses**		**Vet Students**		**Nurse Students**		**Other**	
**Survey Type**	**A**	**B**	**A**	**B**	**A**	**B**	**A**	**B**	**A**	**B**
**Desex Age**										
<1 month	0	0	1	0	0	0	0	1	0	0
1–2 months	1	3	1	4	1	24	0	16	0	0
2–3 months	6	8	15	28	2	40	0	30	1	2
3–4 months	4	14	20	50	5	57	0	50	4	7
>4 months	9	7	78	64	10	63	2	118	1	4
Total *n*	20	32	115	146	18	184	2	215	6	13

A: Face-to-Face Survey.; B: Online Survey.

## Appendix D

Log-linear analysis of three-way interactions between professional role/career length and preferred minimum desexing age of male and female cats.

**Table A4:** Minimum desex ages selected for male cats, and female cats, by veterinary professionals and students. Of 957 surveys, 127 professionals provided no desexing age for male cats, and 141 provided no age for females and were excluded from log-linear analysis (question answered by 87% and 85% of professionals respectively). Two desex age categories were pooled because of small sample sizes (≤1 and 1–2 months). There was a significant three-way interaction between professional role, desex age, and cat sex (G^2^ = 203.60, d.f. = 40, *p* < 0.0001) which remained significant after Bonferroni correction.

***Male Cats***						
	**Minimum Desex Age (months)**					
	<2	2–3	3–4	>4	Other	Total *n*
**Professional Role**						
Veterinarian	7	13	17	15	5	57
Veterinary Nurse	14	62	73	110	51	310
Veterinary Student	41	59	57	53	5	215
Nursing Student	24	42	57	98	7	228
Other	0	6	7	6	1	20
Total *n*	86	182	211	282	69	830
***Female Cats***						
	**Minimum Desex Age (months)**					
	<2	2–3	3–4	>4	Other	Total *n*
**Professional Role**						
Veterinarian	4	14	18	16	5	57
Veterinary Nurse	6	43	70	142	46	307
Veterinary Student	25	42	62	73	6	208
Nursing Student	17	30	50	120	7	224
Other	0	3	11	5	1	20
Total *n*	52	132	211	356	65	816

**Table A5:** Minimum desex ages selected for male cats, and female cats, by veterinarians with different career lengths. Four vets selected no minimum desex age for male or female cats and were excluded from log-linear analysis (question answered by 93% of 61 vets). Two desex age categories were pooled because of small sample sizes (≤1 and 1–2 months). Six career length categories were pooled into three because of small sample sizes (<1 and 1–5 years vs. 6–10 and 11–15 years vs. 16–20 and ≥21 years). There were no significant three-way interactions between career length, desex age, and cat sex (G^2^ = 10.22, d.f. = 22, *p* = 0.98).

***Male Cats***						
	**Minimum Desex Age (months)**					
**Career Length**	<2	2–3	3–4	>4	Other	**Total *n***
<1–5 years	2	3	3	3	1	12
6–15 years	3	1	4	1	1	10
≥16 years	2	8	11	11	3	35
Total *n*	7	12	18	15	5	57
***Female Cats***						
	**Minimum Desex Age (months)**					
**Career Length**	<2	2–3	3–4	>4	Other	**Total *n***
<1–5 years	1	3	3	4	1	12
6–15 years	1	3	4	1	1	10
≥16 years	2	8	11	11	3	35
Total *n*	4	14	18	16	5	57

**Table A6:** Minimum desex ages selected for male cats, and female cats, by veterinary nurses with different career lengths. Three vet nurses did not specify a career length, so their answers were excluded from log-linear analysis. Twelve nurses selected no minimum desexing age for male cats, and 15 selected no age for females (question answered by 96% and 95% of 320 nurses respectively) and were excluded from analysis. Two desex age categories were pooled because of small sample sizes (≤1 and 1–2 months). Six career length categories were pooled into three because of small sample sizes (<1 and 1–5 years vs. 6–10 and 11–15 years vs. 16–20 and ≥21 years). There was no significant three-way interaction between career length, desex age, and cat sex (G^2^ = 27.38, d.f. = 22, *p* = 0.20). There was a single two-way interaction between minimum desex age and cat sex (G^2^ = 11.22, d.f. = 4, *p* = 0.02) but this was insignificant after Bonferroni correction.

***Male Cats***						
	**Minimum Desex Age (months)**					
	<2	2–3	3–4	>4	Other	Total *n*
**Career Length**						
<1–5 years	8	25	25	44	18	120
6–15 years	3	23	28	46	26	126
≥16 years	3	13	20	19	7	62
Total *n*	14	61	73	109	51	308
***Female Cats***						
	**Minimum Desex Age (months)**					
	<2	2–3	3–4	>4	Other	Total *n*
**Career Length**						
<1–5 years	4	20	21	55	19	119
6–15 years	1	12	32	60	19	124
≥16 years	1	10	17	26	8	62
Total *n*	6	42	70	141	46	305

**Table A7:** Minimum desex ages selected for male cats, and female cats, by veterinary science students with different career lengths. Eighty-one vet students selected no minimum desexing age for male cats, and 88 selected no age for females and were excluded from log-linear analysis (question answered by 73% and 70% of 296 students, respectively). Two desex age categories were pooled because of small sample sizes (≤1 and 1–2 months). Several students stated that they had unqualified experience in the veterinary industry, therefore, six career length categories were pooled into two because of small sample sizes (<1 year vs. 1–5, 6–10, 11–15, 16–20, and ≥21 years). There was no significant three-way interaction between career length, desex age, and cat sex (G^2^ = 15.78, d.f. = 13, *p* = 0.26). There was a single two-way interaction between minimum desex age and cat sex (G^2^ = 10.16, d.f. = 4, *p* = 0.04) but this was insignificant after Bonferroni correction.

***Male Cats***						
	**Minimum Desex Age (months)**					
**Career Length**	<2	2–3	3–4	>4	Other	**Total *n***
<1 year	32	50	42	43	4	171
≥1 years	9	9	15	10	1	44
Total *n*	41	59	57	53	5	215
***Female Cats***						
	**Minimum Desex Age (months)**					
**Career Length**	<2	2–3	3–4	>4	**Other**	**Total *n***
<1 year	21	34	44	60	5	164
≥ 1 years	4	8	18	13	1	44
Total *n*	25	42	62	73	6	208

**Table A8:** Minimum desex ages selected for male cats, and female cats, by veterinary nursing students with different career lengths. Twenty-six nursing students selected no minimum desex age for male cats, and 30 selected no age for females and were excluded from log-linear analysis (question answered 90% and 88% of 254 students respectively). Two desex age categories were pooled because of small sample sizes (≤1 and 1–2 months). Several students stated that they had unqualified experience in the veterinary industry, therefore, six career length categories were pooled into two because of small sample sizes (<1 year vs. 1–5, 6–10, 11–15, 16–20, and ≥21 years). There was no significant three-way interaction between career length, desex age, and cat sex (G^2^ = 16.08, d.f. = 13, *p* = 0.24).

***Male Cats***						
	**Minimum Desex Age (months)**					
**Career Length**	<2	2–3	3–4	>4	**Other**	**Total *n***
<1 year	16	24	25	54	4	123
≥1 years	8	18	32	44	3	105
Total *n*	24	42	57	98	7	228
***Female Cats***						
	**Minimum Desex Age (months)**					
**Career Length**	<2	2–3	3–4	>4	**Other**	**Total *n***
<1 year	9	22	24	62	3	120
≥1 years	8	8	26	58	4	104
Total *n*	17	30	50	120	7	224

## Appendix E

Log-linear analysis of three-way interactions between professional role/career length and rationale for preferred minimum desexing age of male and female cats. All answers to two decimal points.

**Table A9:** Weighted frequencies of reasons chosen by different veterinary professionals and students as rationale for choice of minimum desexing ages for male, and female cats. Reasons were provided by 831 respondents for male cats, and 814 for female cats (question answered by 87% and 85% of 957 respondents, respectively). There were significant three-way interactions between professional role, desex reason, and cat sex (G^2^ = 201.64, d.f. = 40, *p* <0.0001), and this remained significant after Bonferroni correction.

***Male Cats***						
	**Reasons for Minimum Desex Age**					
	A.	B.	C.	D.	E.	Total
**Professional Role**						
Veterinarian	13.41	6.57	8.07	19.41	9.50	56.96
Veterinary Nurse	77.15	16.31	71.00	83.01	62.21	309.68
Veterinary Student	46.58	39.45	56.68	64.51	7.48	214.70
Nursing Student	68.33	16.38	57.01	78.16	8.83	228.71
Other	4.49	0.00	4.49	8.16	2.83	19.97
Total	209.96	78.71	197.25	253.25	90.85	830.02
***Female Cats***						
	**Reasons for Minimum Desex Age**					
	A.	B.	C.	D.	E.	Total
**Professional Role**						
Veterinarian	12.91	7.07	8.07	16.41	12.5	56.96
Veterinary Nurse	87.21	16.63	58.24	81.73	62.8	306.61
Veterinary Student	45.05	43.16	50.64	60.3	6.4	205.55
Nursing Student	67.15	14.89	59.51	77.99	4.16	223.70
Other	3.66	0.00	4.66	7.83	3.83	19.98
Total	215.98	81.75	181.12	244.26	89.69	812.80

A: Respondent believed there is less anaesthetic risk at the selected preferred age. B: Respondent had limited experience with desexing cats younger than the selected preferred age. C: Respondent had concerns about normal development of anatomy/physiology/behaviour in cats desexed younger than the selected preferred age. D: Respondent believed desexing at the selected preferred age will help reduce the number of stray/abandoned/feral cats in their community. E: Respondent provided alternative reason/s to justify the selected preferred age.

**Table A10:** Weighted frequencies of reasons chosen by veterinarians as rationale for minimum desex ages of male and female cats. Reasons were provided by 57 vets for both cat sexes (question answered by 93% of 61 vets). Six career length categories were pooled into three because of small sample sizes (<1 and 1–5 years vs. 6–10 and 11–15 years vs. 16–20 and ≥21 years). There was no significant three-way interaction between career length, desex reason, and cat sex (G^2^ = 20.36, d.f. = 22, *p* = 0.56).

***Male Cats***						
	**Reasons for Minimum Desex Age**					
	A.	B.	C.	D.	E.	Total
0–5 years	3.25	2.91	2.91	2.91	0.00	11.98
6–15 years	2.50	1.00	0.50	5.50	0.50	10.00
≥16 years	7.66	2.66	6.66	10.00	8.00	34.98
Total	13.41	6.57	10.07	18.41	8.50	56.96
***Female Cats***						
	**Reasons for Minimum Desex Age**					
	A.	B.	C.	D.	E.	Total
0–5 years	3.25	2.91	0.91	2.41	2.50	11.98
6–15 years	2.50	1.00	0.50	5.50	0.50	10.00
≥16 years	7.16	3.16	6.66	8.50	9.50	34.98
Total	12.91	7.07	8.07	16.41	12.50	56.96

A: Respondent believed there is less anaesthetic risk at the selected preferred age. B: Respondent had limited experience with desexing cats younger than the selected preferred age. C: Respondent had concerns about normal development of anatomy/physiology/behaviour in cats desexed younger than the selected preferred age. D: Respondent believed desexing at the selected preferred age will help reduce the number of stray/abandoned/feral cats in their community. E: Respondent provided alternative reason/s to justify the selected preferred age.

**Table A11:** Weighted frequencies of reasons chosen by veterinary nurses as rationale for minimum desex ages of male and female cats. Reasons were provided by 308 nurses for male cats, and 305 nurses for female cats (question answered by 96% and 95% of 320 nurses, respectively). Six career length categories were pooled into three because of small sample sizes (<1 and 1–5 years vs. 6–10 and 11–15 years vs. 16–20 and ≥21 years). There was no significant three-way interaction between career length, desex reason, and cat sex (G^2^ = 18.64, d.f. = 22, *p* = 0.67). There was a single significant two-way interaction between career length and minimum desex age (G^2^ = 16.66, d.f. = 8, *p* = 0.03).

***Male Cats***						
	**Reasons for Minimum Desex Age**					
	A.	B.	C.	D.	E.	Total
0–5 years	32.31	9.99	21.48	30.15	25.99	119.92
6–15 years	29.62	4.82	29.11	37.13	25.15	125.83
≥16 years	14.72	1.50	19.39	15.73	10.57	61.91
Total	76.65	16.31	69.98	83.01	61.71	307.66
***Female Cats***						
	**Reasons for Minimum Desex Age**					
	A.	B.	C.	D.	E.	Total
0–5 years	36.95	9.98	17.63	28.46	25.82	118.84
6–15 years	33.79	4.32	22.8	37.13	25.82	123.86
≥16 years	15.64	2.00	17.81	15.81	10.66	61.92
Total	86.38	16.30	58.24	81.40	62.30	304.62

A: Respondent believed there is less anaesthetic risk at the selected preferred age. B: Respondent had limited experience with desexing cats younger than the selected preferred age. C: Respondent had concerns about normal development of anatomy/physiology/behaviour in cats desexed younger than the selected preferred age. D: Respondent believed desexing at the selected preferred age will help reduce the number of stray/abandoned/feral cats in their community. E: Respondent provided alternative reason/s to justify the selected preferred age.

**Table A12:** Weighted frequencies of reasons for selecting specific desex ages for male and female cats by veterinary science students of different career lengths. Reasons were provided by 215 veterinary students for male cats, and 206 for female cats (question answered by 73% and 70% of 296 students, respectively). Several students stated that they had unqualified experience in the veterinary industry, therefore, six career length categories were pooled into two because of small sample sizes (<1 year vs. 1–5, 6–10, 11–15, 16–20, and ≥21 years). There was no significant three-way interaction between career length, desex reason, and cat sex (G^2^ = 6.20, d.f. = 13, *p* = 0.94).

***Male Cats***						
	**Reasons for Minimum Desex Age**					
	A.	B.	C.	D.	E.	Total
**Career Length**						
< 1 year	36.84	33.96	43.19	50.85	4.90	169.74
≥1 years	10.74	6.49	13.49	11.66	2.58	44.96
Total	47.58	40.45	56.68	62.51	7.48	214.70
***Female Cats***						
	**Reasons for Minimum Desex Age**					
	A.	B.	C.	D.	E.	Total
**Career Length**						
< 1 year	34.90	37.09	39.24	48.06	3.32	162.61
≥1 years	10.15	6.07	11.40	12.24	3.08	42.94
Total	45.05	43.16	50.64	60.30	6.40	205.55

A: Respondent believed there is less anaesthetic risk at the selected preferred age. B: Respondent had limited experience with desexing cats younger than the selected preferred age. C: Respondent had concerns about normal development of anatomy/physiology/behaviour in cats desexed younger than the selected preferred age. D: Respondent believed desexing at the selected preferred age will help reduce the number of stray/abandoned/feral cats in their community. E: Respondent provided alternative reason/s to justify the selected preferred age.

**Table A13:** Weighted frequencies of reasons for selecting specific desex ages for male and female cats by veterinary nursing students of different career lengths. Reasons were provided by 229 nursing students for male cats, and 224 for female cats (question answered by 90% and 88% of 254 students, respectively). Several students stated that they had unqualified experience in the veterinary industry, therefore, six career length categories were pooled into two because of small sample sizes (<1 year vs. 1–5, 6–10, 11–15, 16–20, and ≥21 years). There was no significant three-way interaction between career length, desex reason, and cat sex (G^2^ = 4.64, d.f. = 13, *p* = 0.98).

***Male Cats***						
	**Reasons for Minimum Desex Age**					
	A.	B.	C.	D.	E.	Total
<1 year	37.62	6.82	34.12	40.78	5.50	124.84
≥1 years	30.71	9.56	22.89	37.38	3.33	103.87
Total	68.33	16.38	57.01	78.16	8.83	228.71
***Female Cats***						
	**Reasons for Minimum Desex Age**					
	A.	B.	C.	D.	E.	Total
<1 year	36.88	7.58	31.88	41.55	3.00	120.89
≥1 years	30.27	7.31	27.63	36.44	1.16	102.81
Total	67.15	14.89	59.51	77.99	4.16	223.70

A: Respondent believed there is less anaesthetic risk at the selected preferred age. B: Respondent had limited experience with desexing cats younger than the selected preferred age. C: Respondent had concerns about normal development of anatomy/physiology/behaviour in cats desexed younger than the selected preferred age. D: Respondent believed desexing at the selected preferred age will help reduce the number of stray/abandoned/feral cats in their community. E: Respondent provided alternative reason/s to justify the selected preferred age.
